# Transcriptome Responses of Ripe Cherry Tomato Fruit Exposed to Chilling and Rewarming Identify Reversible and Irreversible Gene Expression Changes

**DOI:** 10.3389/fpls.2021.685416

**Published:** 2021-07-16

**Authors:** Donald A. Hunter, Nathanael J. Napier, Zoe A. Erridge, Ali Saei, Ronan K. Y. Chen, Marian J. McKenzie, Erin M. O’Donoghue, Martin Hunt, Laurie Favre, Ross E. Lill, David A. Brummell

**Affiliations:** ^1^The New Zealand Institute for Plant & Food Research Limited, Food Industry Science Centre, Palmerston North, New Zealand; ^2^Centre for Postharvest and Refrigeration Research, Massey University, Palmerston North, New Zealand

**Keywords:** tomato (*Solanum lycopersicum*), chilling injury, fruit softening, cell wall modifying genes, heat shock protein genes

## Abstract

Tomato fruit stored below 12°C lose quality and can develop chilling injury upon subsequent transfer to a shelf temperature of 20°C. The more severe symptoms of altered fruit softening, uneven ripening and susceptibility to rots can cause postharvest losses. We compared the effects of exposure to mild (10°C) and severe chilling (4°C) on the fruit quality and transcriptome of ‘Angelle’, a cherry-type tomato, harvested at the red ripe stage. Storage at 4°C (but not at 10°C) for 27 days plus an additional 6 days at 20°C caused accelerated softening and the development of mealiness, both of which are commonly related to cell wall metabolism. Transcriptome analysis using RNA-Seq identified a range of transcripts encoding enzymes putatively involved in cell wall disassembly whose expression was strongly down-regulated at both 10 and 4°C, suggesting that accelerated softening at 4°C was due to factors unrelated to cell wall disassembly, such as reductions in turgor. In fruit exposed to severe chilling, the reduced transcript abundances of genes related to cell wall modification were predominantly irreversible and only partially restored upon rewarming of the fruit. Within 1 day of exposure to 4°C, large increases occurred in the expression of alternative oxidase, superoxide dismutase and several glutathione S-transferases, enzymes that protect cell contents from oxidative damage. Numerous heat shock proteins and chaperonins also showed large increases in expression, with genes showing peak transcript accumulation after different times of chilling exposure. These changes in transcript abundance were not induced at 10°C, and were reversible upon transfer of the fruit from 4 to 20°C. The data show that genes involved in cell wall modification and cellular protection have differential sensitivity to chilling temperatures, and exhibit different capacities for recovery upon rewarming of the fruit.

## Introduction

Tomato is an originally tropical fruit that requires postharvest storage above 12°C to avoid deleterious effects on flavor (Maul et al., 2000). Storage at lower temperatures is used to prolong storage life and enable shipping to more distant markets, but can result in severe chilling injuries including surface pitting, uneven or partial ripening, reduced softening, accelerated softening, mealiness, water-soaking, and susceptibility to rots (Cheng and Shewfelt, 1988; Jackman et al., 1992; Lurie and Sabehat, 1997; Devaux et al., 2005; Vega-Garcia et al., 2010; Rugkong et al., 2011; Biswas et al., 2012; Albornoz et al., 2019). Chilling injuries are thought to result initially from phase transitions of cell membranes and increases in reactive oxygen species (ROS) such as superoxide radical, hydrogen peroxide and hydroxyl radical, which if not detoxified cause inactivation of enzymes, degradation of proteins and DNA, and lipid peroxidation that compromises membrane function (Sevillano et al., 2009). Membrane deterioration can lead to impaired ATP biosynthesis, reduced respiration, and leakage of ions, metabolites and water between cell compartments or out of the cell, and even cell rupture.

Tomato is a well-established model system for understanding ripening, but less is known about its responses to stress. The susceptibility of tomato fruit to chilling injury varies with cultivar and ripening stage. Frequently the most severe symptoms appear when green or breaker fruit are transferred to shelf temperatures of 20–22°C for ripening, after storage below approximately 6°C for 2 weeks or more (Biswas et al., 2016). If ripening is already advanced prior to cold storage, fruit are more resistant to chilling stress, display fewer chilling injury symptoms, and can be stored at lower temperatures and for longer periods than mature green or breaker fruit (Hobson, 1987; Gómez et al., 2009). Previous studies of transcriptome responses to chilling stress in tomato have used fruit harvested and stored at the mature-green or breaker stages, where ripening processes form a major part of transcriptional activity (Cruz-Mendívil et al., 2015; Albornoz et al., 2019; Zhang et al., 2019; Tang et al., 2020). The transcriptome responses to chilling stress of cherry tomato fruit that have already achieved full ripeness prior to cold storage are currently unknown.

Ripening in tomato involves changes in gene expression controlling accumulation of the carotenoid lycopene, alterations to sugar and organic acid composition, production of aroma volatiles, and changes to fruit firmness and textural properties. Chilling injury affects the gene expression of many of these aspects of the ripening program (Zhang et al., 2019; Tang et al., 2020), as well as the ethylene signaling and response pathway (Rugkong et al., 2011). Fruit softening during normal ripening is caused by reductions in cell turgor (Shackel et al., 1991) and by a range of modifications to the cell wall and middle lamella brought about by enzymes secreted from the symplast into the apoplast (Brummell and Harpster, 2001). Cell wall changes include depolymerization of homogalacturonan and hemicelluloses, demethylesterification of homogalacturonan, solubilization of pectins and hemicelluloses, and loss of pectic β-galactan and α-arabinan side chains (Brummell, 2006). Together, these changes alter polysaccharide structure and the bonding between polymers in the wall, causing wall swelling and reducing wall strength and intercellular adhesion. The enzymes involved are encoded by ripening-related members of gene families including polygalacturonase (PG), pectate lyase (PL), pectin methylesterase (PME), expansin (EXP), endo-1,4-β-glucanase (“cellulase,” Cel), xyloglucan endo-transglycosylase/hydrolase (XTH), β-galactosidase and α-arabinofuranosidase.

An extended storage life enables produce to be shipped by sea rather than by air, but after cold storage fruit need to possess an acceptable shelf-life at 20°C. In this study, we examined the effects of long-term mild and severe chilling on ripe fruit of a relatively chilling-resistant cultivar, ‘Angelle’, of the cherry tomato type. This cultivar is commercially harvested at the red ripe stage and shows extensive postharvest storage life, even at 20°C. Two storage temperatures, 10 and 4°C, were chosen as a way to separate gene expression responses to mild and severe cold stress, and to investigate which gene expression changes were reversible upon rewarming of the fruit. Since fruit were already ripe at harvest and exhibiting full color, genes related to fruit softening and responses to oxidative and abiotic stress, rather than carotenoid accumulation or ripening transcription factors, were chosen for examination during cold storage.

## Materials and Methods

### Fruit Material

Fruit of cherry tomato (*Solanum lycopersicum* var. *cerasiforme* L.) ‘Angelle’ were obtained from a packhouse at commercial ripeness during the middle of the production season in 2018 and 2019. Plastic clamshell punnets each containing 20–23 fruit arrived at the research facility the day after packing, and were stored overnight at 20°C to ensure evaporation of surface condensation moisture.

In each of the two experiments, one set of replicates was processed after the overnight storage at 20°C (termed Day 0). In the first experiment (year 2018, total of 30 punnets), storage at three different temperatures was compared. One set of punnets was maintained at 20°C for 6 days to study development at a typical shelf temperature. Half the remaining punnets were placed in cold storage at 10°C and the other half at 4°C, representing mild and severe chilled storage conditions, respectively. Samples were removed at 6 and 27 days, with further sets of replicates stored for 27 days plus an additional 1 and 6 days at 20°C. In the second experiment (year 2019, total of 21 punnets), the focus was on earlier molecular responses to chilling temperature. Punnets were stored at 4°C, with replicates removed after 1, 4, 12, and 19 days. Additional replicates were removed at 12 and 19 days and transferred to 20°C for 7 days. A schematic representation of the experimental design is shown in Supplementary Figure 1. At each temperature/time point, analysis was carried out using three biological replicates (punnets of fruit).

### Physiological Measurements

Firmness was measured on a TA.XT Plus Texture Analyser (Stable Micro Systems, Godalming, United Kingdom) with a 75-mm compression plate attachment. The force required for compression of 0.5 mm was measured at a test speed of 0.5 mm s^–1^, with a trigger force of 10 g. Three biological replicates were analyzed at each time point, comprising eight fruit per biological replicate punnet. Three independent firmness measurements were taken at different locations on each fruit, avoiding the locule wall (*n* = 72).

Free juice was measured using a modification of the method developed for peach by Lill and van der Mespel (1988). Free juice was estimated in fruit stored at 20°C for 6 days (never exposed to cold), and for fruit stored for 27 days at 4 or 10°C plus 20°C for 6 days. For each fruit, a shallow cut was made in the skin in the shape of a 1-cm square, the peel carefully removed and the underlying pericarp excised. An identical piece of pericarp was excised from the opposite side of the same fruit. The two pericarp pieces were blotted dry of surface juice and locule gel then both placed in a 5-mL disposable syringe (Terumo, Somerset, NJ, United States) with luer taper (aperture ∼1.5 mm) but without a needle. The tissue (∼0.9–1.4 g) was pushed through the syringe into a weighed microfuge tube, which was re-weighed then the homogenate was centrifuged at 13,000*g* for 5 min. The supernatant was transferred to a new weighed tube, and the weight of the supernatant calculated as a percentage of the weight of the homogenate. Six fruit per treatment were assessed, two from each of three different replicate punnets.

### RNA Isolation, RNA-Seq Library Construction and Sequencing

Fruit pericarp tissue was separated from locule walls and seeds, then the pericarp was snap frozen in liquid nitrogen. For each temperature/time point there were three biological replicates (punnets), each consisting of four pooled fruit. The tissue was ground to a fine powder under liquid nitrogen conditions using an analytical grinding mill. RNA was isolated using a modified CTAB method (Gambino et al., 2008), using aliquots of 300 mg of ground tissue in 900 μL of CTAB buffer. Final RNA pellets were washed with 70% ethanol and resuspended in 33 μL of sterile water. RNA samples were quantified using a NanoDrop 1000 Spectrophotometer (Thermo Fisher Scientific, Auckland, New Zealand) and RNA integrity assessed using a Standard Sensitivity RNA Analysis Kit (DNF-471) on a Fragment Analyzer 5300 System (Agilent Technologies, Santa Clara, CA, United States). All samples had an *A*_260/280_ ≥1.8 and an RNA Integrity Number ≥8. Samples for Experiment 1 were sent to the Australian Genome Research Facility (Melbourne, Australia) for library preparation and 100 bp paired end sequencing on a NovaSeq platform. Samples for Experiment 2 were sent via Custom Science (Auckland, New Zealand) to a sequencing center for stranded RNA library preparation and 150 bp paired end sequencing on a HiSeq platform.

### RNA-Seq Analysis

Raw read quality was assessed using FastQC software^1^. SortMeRNA (Kopylova et al., 2012) was used to filter rRNA from the raw reads. Trimmomatic (Bolger et al., 2014) with settings “HEADCROP:10 SLIDINGWINDOW:5:20 MINLEN:50” was used to trim the low quality reads and sequencing adapters. The trimmed reads were mapped to the *S. lycopersicum* build_3.00 genome using STAR (Dobin et al., 2013) with default parameters. HTSeq-count supplied with ITAG3.2 gene models was used to compute the number of raw reads mapped to each gene.

For differential expression analysis, input genes were initially filtered to remove genes with mean counts of less than 200 across all samples. Gene counts of filtered reads were normalized using the Relative Log Expression method of DESeq2 with default parameters (Love et al., 2014).

The pairwise gene selection method was used to select the top 50 most variable genes per pairwise comparison, and principal component analysis (PCA) performed using the FactoMineR package^2^. Genes for cluster analysis (displayed in the expression pattern figures) were first identified as having a fold change of >3-fold up or down and *P*-value < 0.01. They then underwent variance stabilizing transformation and hierarchical clustering using the “complete” clustering and “euclidean” distance methods. For heat maps, coloration was performed in Excel (Microsoft, Seattle, WA, United States) using conditional formatting and the red, yellow, green color scale with red for the highest transcript abundance. The conditional formatting was performed on each row separately so that the highest and lowest values for each transcript were colored red and green, respectively.

Enriched biological themes and KEGG pathways were identified using the DAVID Gene Functional Classification Tool^3^ (Huang et al., 2009) with a >2-fold change criteria for the gene lists. Gene stable IDs in the gene list were first converted to UniProtKB/TrEMBL IDs at Biomart^4^ and used as input into DAVID where they were converted to Entrez IDs for annotation enrichment analysis. The analysis identified enrichment in the gene sets of KEGG pathways^5^, and Functional Annotational Clustering was used to identify further enriched biological themes within the gene sets.

## Results

### Characterization of Chilling Effects

**FIGURE 1:**
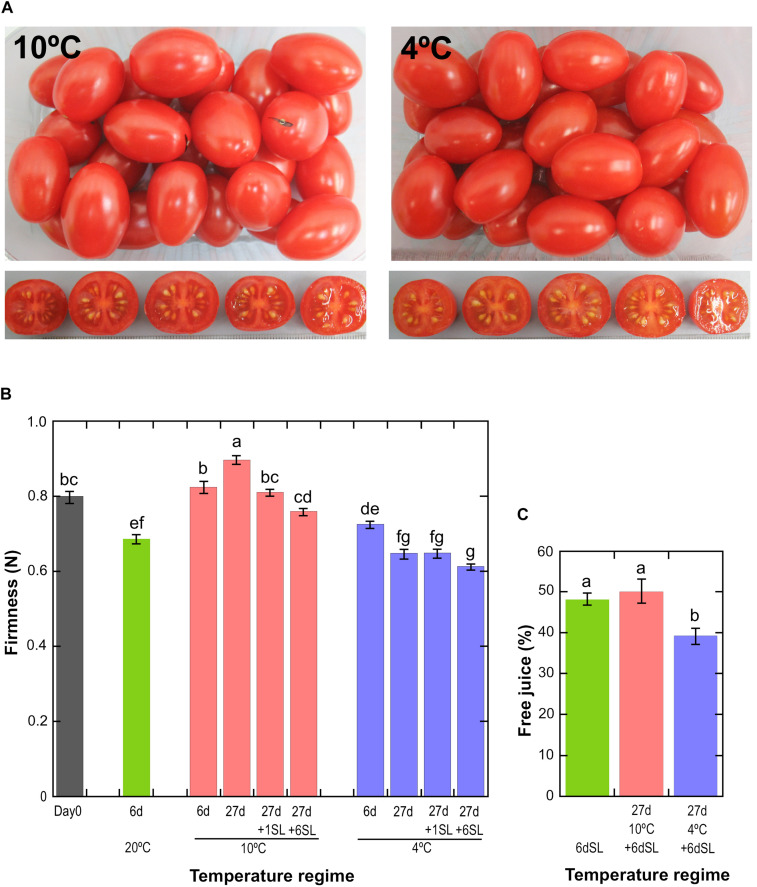
Physiological effects of storage at different temperatures. **(A)** Fruit appearance after storage at 10 or 4°C for 27 days, followed by an additional 6 days at a shelf temperature of 20°C. **(B)** Mean fruit firmness ± SE (*n* = 72) measured by compression. Storage at 20°C for 6 days, or 10 or 4°C for up to 27 days with or without an additional shelf-life (SL) period of 1 or 6 days. Means not sharing a common letter are significantly different between groups at *P* = 0.05 as determined by LSD after a one-way ANOVA. **(C)** Mean pericarp free juice ± SD (*n* = 6) as determined using a homogenate and centrifugation method. Fruit were stored for 6 days at 20°C, without or with prior storage for 27 days at 10 or 4°C. Means not sharing a common letter are significantly different between groups at *P* = 0.001 as determined by LSD after a one-way ANOVA.

Ripe tomato fruit were exposed to either 10°C or 4°C chilled storage conditions for various times to discover the effects of mild and severe temperature stress on gene expression and chilling injury. Fruit were fully ripe at harvest, as shown by measurements of fruit color, which did not increase in redness relative to Day 0 during storage at either 20 or 10°C (Supplementary Figure 2). The slight reduction in the redness of fruit stored at 4°C is a mild chilling injury (Supplementary Figure 2). Even after storage for 27 days at 10 or 4°C followed by an additional 6 days at shelf-life conditions of 20°C, ripe ‘Angelle’ fruit did not display the visible symptoms of chilling injury often seen in unripe cold-stored fruit such as pitting, shriveling, soft patches or rots (Figure 1A). However, textural deterioration in the form of differential softening and mealiness in response to storage at different temperatures was observed. Storage of fruit for 6 days at three different temperatures showed that fruit stored at 10°C did not soften significantly relative to Day 0, whereas fruit stored at 20 or 4°C did soften (Figure 1B). Fruit stored at 10°C for 27 days actually tended to increase in firmness, before softening after transfer to shelf-life conditions of 20°C. Fruit stored at 4°C for 27 days softened considerably in comparison to fruit stored at 10°C, and this accelerated softening is a well-known chilling damage (Jackman et al., 1992). Transition of fruit stored at 4°C to a shelf temperature of 20°C induced little additional loss of firmness, suggesting that ripening-related softening barely resumed upon transfer to warmer temperatures.

A common symptom of chilling injury in fleshy fruit is the development of mealiness, which is caused by alterations in cell wall metabolism that affect the properties of pectin, resulting in the binding of free water and a higher degree of cell separation that causes reduced cell breakage upon biting and chewing (Lurie and Crisosto, 2005). The condition is characterized by a dry texture and lack of free juice, but is not due to whole-fruit water loss. Mealiness is the major chilling injury symptom in cold-stored peaches (Lurie and Crisosto, 2005) but tomatoes are also susceptible (Jackman et al., 1992), and the condition can be assessed by measuring free juice (Figure 1C). Fruit stored for 6 d at 20°C (never exposed to cold) or stored for 27 days at 10°C followed by 6 days at 20°C yielded approximately 50% free juice, but this was reduced to 40% if fruit had been stored at 4°C. This shows that a degree of mealiness had developed during storage at severe chilling temperature. There were no significant differences in whole-fruit weight loss between the different storage regimes (Supplementary Figure 3).

### Expression Patterns in Response to Mild or Severe Chilling Stress

Transcriptome profiling by RNA-Seq was performed after various periods of exposure to mild or severe chilling temperature, followed in some cases by a recovery period at 20°C. Sequencing depth was good, with an average of 18.5 million uniquely mapped reads per sample in Experiment 1 and 13.9 million in Experiment 2 (Supplementary Table 1).

#### i) Transcriptional dynamics during mild and severe chilling

**FIGURE 2:**
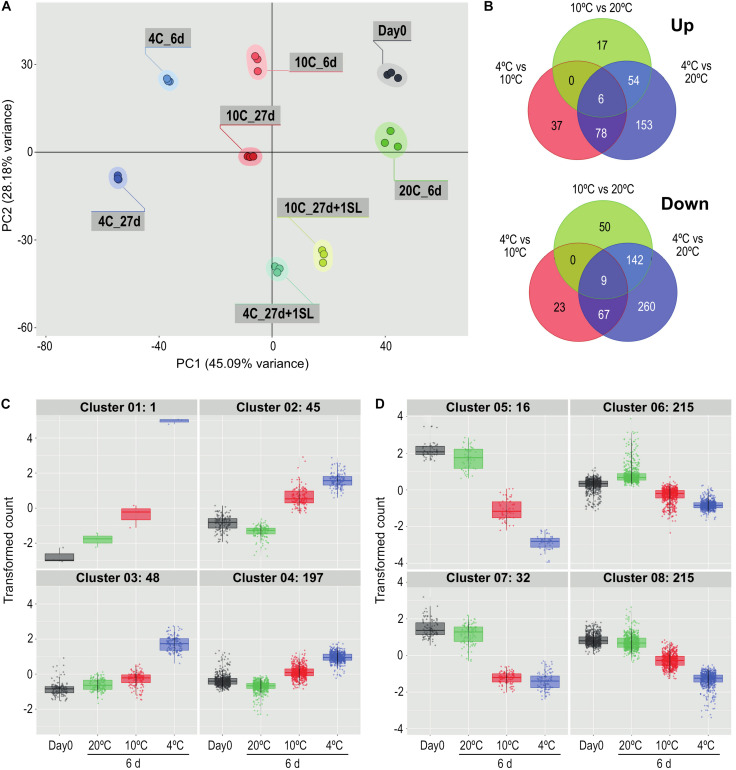
Comparison of the effects of storage at different temperatures on the ripe tomato fruit transcriptome. **(A)** Principal component analysis of transcriptome profiles based on the 50 most variable genes in each pairwise comparison (total of 720 most variable genes), showing changes during storage at 4, 10, or 20°C. An additional 1-day period at a shelf-life (SL) temperature of 20°C was also included after 27 days. **(B)** Venn diagrams showing numbers of genes differentially expressed at 4, 10, and 20°C at 6 days of storage. For each pairwise comparison, the count values in the lower temperature treatment were compared with those in the higher temperature treatment. **(C,D)** Hierarchical cluster analysis of the gene sets that were up-regulated (291) and down-regulated (478) from the entire blue circles of **(B)** obtained from the 4°C versus 20°C comparison. Expression patterns were clustered into four groups each for up-regulated **(C)** and down-regulated **(D)** genes. Genes were considered differentially expressed if they had a mean count in all compared samples of 200 or more, an adjusted *P*-value of 0.01 and >3-fold increase or decrease.

In the first experiment, tomato fruit were exposed to 20°C for 6 days, or 10°C or 4°C for 6 or 27 days. PCA of the 720 most variable genes (derived from the 50 most variable genes in each of the pairwise comparisons) showed that the three replicates of each treatment clustered closely together (Figure 2A). This indicated that the transcriptome response of the biological replicates was consistent for each of the treatments. The majority of the dataset variance was explained by Component 1 (45.09% of the variance) and was associated with the temperature response, with increasing storage time and decreasing temperature shifting the samples further from the Day 0 control. Conversely, returning the stored fruit to 20°C shifted the cold-stored samples closer to the Day 0 samples, suggesting that the transcriptome response to temperature was in part reversible. Less of the variance (28.18%) was explained by Component 2, and this appeared to be associated with the progression of development, since the samples held at 20°C for 6 days were further away from Day 0 samples on this axis, but not on the Component 1 axis.

Comparisons at 6 days among the three different storage temperatures found that, relative to 20°C, fewer genes were up-regulated by exposure to 4°C than were down-regulated (291 versus 478 using a >3-fold change criterion, Figure 2B). Of the 291 genes that were up-regulated, 153 were chilling-specific and showed altered expression only at 4°C. Of the 478 genes that were down-regulated at 4°C, 260 were chilling-specific. The number of genes specifically up- or down-regulated at 4 vs 20°C far exceeded the numbers with altered expression specifically at 4 vs 10°C or 10 vs 20°C. Only 15 genes (six up-regulated and nine down-regulated, 1.67% of the total) showed altered gene expression in all three temperature comparisons.

The 291 genes up-regulated and 478 genes down-regulated at 4°C were categorized into four up-regulated and four down-regulated expression patterns by hierarchical clustering (Figures 2C,D). The number of genes in the clusters ranged from 1 to 215, and the genes comprising each cluster are listed in Supplementary Table 2. Relative to storage for an equivalent time at 20°C, the largest increase in transcript abundance specifically in response to chilling temperature was the single gene in Cluster 1 (Figure 2C), encoding a Small Auxin Up-Regulated (SAUR)-like auxin-responsive family protein (Solyc06g053260.1.1), whose transcript abundance was 190-fold higher at 4°C but only four-fold higher at 10°C. Genes with a similar but less extreme expression pattern in Cluster 3 included an alternative oxidase (Solyc08g075540.4.1), a glutathione S-transferase (Solyc12g011310.2.1) and two Dicer-like ribonucleases (Solyc11g008530.2.1, Solyc11g008540.2.1). The 45 genes in Cluster 2 had similar transcript abundances at both 4 and 10°C, and showed an increase partly because transcript abundance at 20°C had declined. Cluster 4 contained genes with only a moderate increase at 4°C. In contrast, the transcript abundance of many genes was negatively affected by chilling temperatures (Figure 2D). In Cluster 5, expression of 16 genes was dramatically down-regulated (some by >1000-fold) at 4°C, but were also significantly down-regulated at 10°C. This group included the ripening-related cell wall-modifying genes *EXP1*, *XTH5* and *Cel2*. Genes in Cluster 7 were affected similarly by 4 and 10°C, and included *PME2*. The vast majority of down-regulated genes were in Clusters 6 and 8, showing moderate reduction in transcript abundance at 4°C and also some reduction at 10°C. Cluster 8 included β-galactosidase *TBG4* and *XYL1*.

#### ii) Rapidity and reversibility of severe chilling-induced expression changes

**FIGURE 3:**
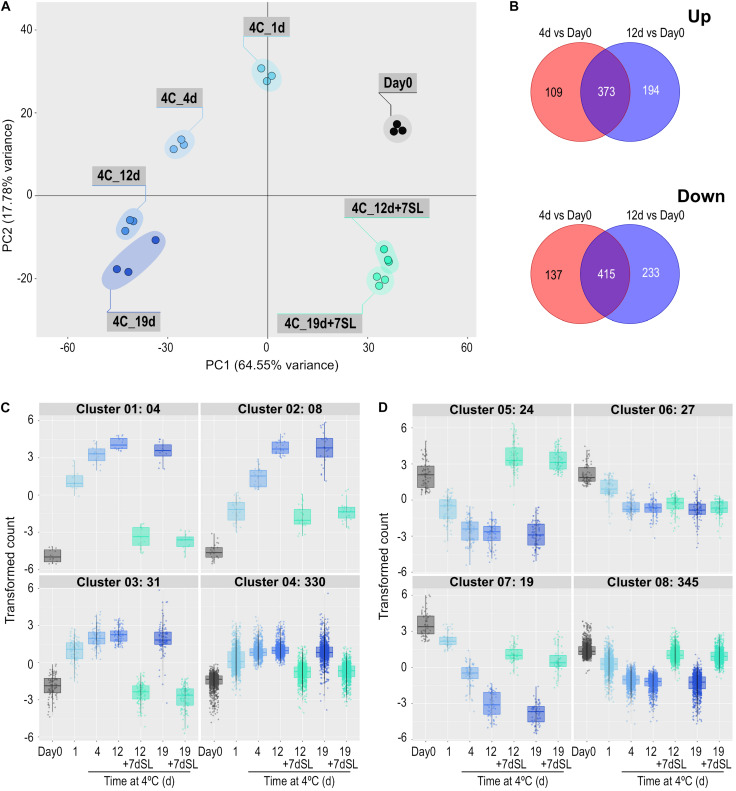
Effect of storage at 4°C for various times on the ripe tomato fruit transcriptome. An additional 7-day period at a shelf-life (SL) temperature of 20°C was also included after 12 and 19 days. **(A)** Principal component analysis of transcriptome profiles based on the 50 most variable genes in each pairwise comparison (total of 617 most variable genes). **(B)** Venn diagrams showing numbers of genes differentially expressed at 4 and 12 days of exposure to 4°C, relative to Day 0. **(C,D)** Hierarchical cluster analysis of the gene sets that were up-regulated (373) and down-regulated (415) at both time points compared with Day 0. Expression patterns were clustered into four groups each for up-regulated **(C)** and down-regulated **(D)** genes. Genes were considered differentially expressed if they had a mean count in all compared samples of 200 or more, an adjusted *P*-value of 0.01 and >3-fold increase or decrease.

In Experiment 2, ‘Angelle’ fruit were exposed to 4°C for 1, 4, 12, and 19 days, with an additional storage period of 7 days at 20°C after the latter two times. This experiment focused on the rapidity of transcriptome responses to chilling and the potential for recovery at a shelf temperature of 20°C. PCA analysis confirmed that all replicates grouped together, and that exposure to chilling temperature caused substantial shifts to the left in Component 1 (representing 64.55% of the variance) (Figure 3A). Movement was relatively large at 1 and 4 days, less so at 12 days and no further shift to the left occurred at 19 days. Between 1 and 19 days there was a shift down in Component 2 (representing 17.78% of the variance). An additional 7 days at shelf temperature after cold storage for 12 or 19 days caused a shift to the right in Component 1, almost back to the equivalent position of Day 0 prior to cold exposure, but still separated from Day 0 in Component 2. PCA analysis of the combined data from both seasons showed largely harmonious agreement between the two experiments (Supplementary Figure 4).

Differentially expressed genes were determined at 4 and 12 days of storage at chilling temperature, relative to expression at Day 0. Some genes were differentially expressed at only one of the two times and these were eliminated to concentrate on the 373 genes up-regulated and 415 genes down-regulated at both times (Figure 3B). The expression patterns of these gene sets were again split into four up-regulated and four down-regulated groups based on hierarchical clustering (Figures 3C,D and Supplementary Table 3). For up-regulated genes, the number of genes in the clusters varied between 4 and 330 (Figure 3C). Cluster 1 contained the genes that showed the most rapid and largest increase in transcript abundance at 4°C, and whose transcript abundance returned to close to pre-exposure values upon rewarming. This cluster included the SAUR-like auxin-responsive family protein (Solyc06g053260.1.1) highlighted in Experiment 1. Clusters 2–4 contained genes with a similar expression profile to Cluster 1, but were less dramatically increased or were not able to return to Day 0 values after rewarming. Cluster 4 contained numerous genes involved in amelioration of stress, including an alternative oxidase (Solyc08g075540.4.1), a superoxide dismutase (Solyc09g082690.3.1), four glutathione S-transferases, and numerous heat shock proteins (HSPs) and chaperonin proteins. Relative to Day 0, a large number of genes were progressively down-regulated during chilling (Figure 3D). Those in Cluster 5 increased to above the Day 0 value upon subsequent transfer to shelf temperature, those in Clusters 7 and 8 were able to return partially or almost completely to pre-exposure mRNA abundances upon rewarming, while those in Cluster 6 remained mostly depressed. Several genes putatively involved in ripening-related cell wall modification were in these clusters: *XTH5* was in Cluster 5, pectate lyase *PL* in Cluster 6, *EXP1*, *Cel2*, and *PME2* in Cluster 7, and *TBG4* and *PME1.9* in Cluster 8. The ripening-related transcription factor *COLORLESS NON-RIPENING* (*CNR*) was also in Cluster 8.

### Effects of Chilling on Transcripts Encoding Genes Related to Cell Wall Modification

**FIGURE 4:**
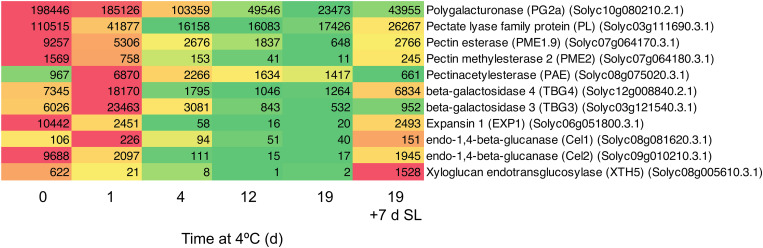
Heat map showing cell wall modification-related genes differentially expressed in response to storage at 4°C. An additional 7-day period at a shelf-life (SL) temperature of 20°C was also included after 19 days. Red and green colors represent high and low mRNA abundance, respectively, within each transcript. Numbers in the boxes are DESeq2 mean normalized counts using the median of ratios method.

Since both accelerated softening and the development of mealiness may be related to cell wall metabolism, the expression of genes encoding enzymes involved in cell wall modification was examined in detail. In general, genes involved in cell wall modification were down-regulated in response to chilling (Figure 4). After 1 day of exposure to 4°C, some genes were slow to change in mRNA abundance (*PG2a*), some declined substantially (including *PL, EXP1, Cel2*), while others transiently increased (including *PAE, TBG3, TBG4*). However, with the exception of *PAE* which showed the opposite trend, by 4 days of exposure all were substantially lower than at Day 0 and remained so until 19 days. Upon the fruit returning to 20°C for 7 days, all of the genes with reduced mRNA abundance showed some increase, but only *TBG4*, *Cel1*, and *XTH5* achieved values close to or above the values at Day 0. This suggests that the ripening-related cell wall-modification program only partially resumed during rewarming to 20°C.

### Effects of Chilling on Transcripts Encoding Genes Related to Stress Amelioration

**FIGURE 5:**
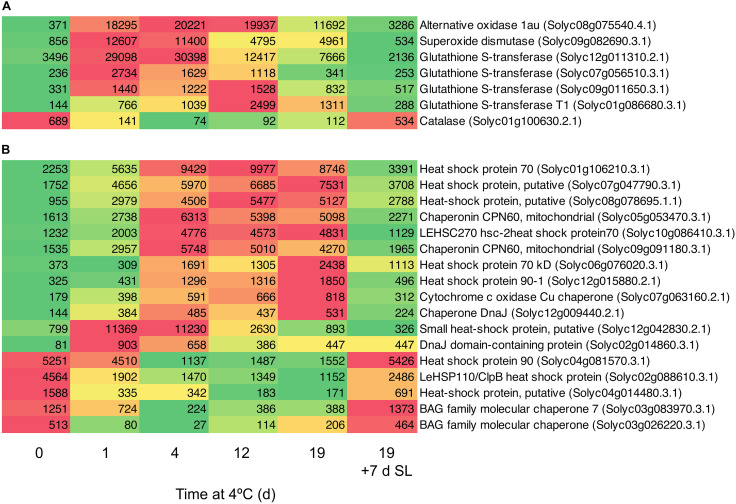
Heat maps showing stress-related genes differentially expressed in response to storage at 4°C. An additional 7-day period at a shelf-life (SL) temperature of 20°C was also included after 19 days. **(A)** Genes encoding enzymes involved in oxidative stress. **(B)** Genes encoding heat shock and molecular chaperone proteins. Red and green colors represent high and low mRNA abundance, respectively, within each transcript. Numbers in the boxes are DESeq2 mean normalized counts using the median of ratios method.

A number of genes known to be involved in combating abiotic stress were up-regulated in response to chilling exposure. These included several genes encoding enzymes involved in scavenging ROS (Figure 5A). Within 1 day, the transcript abundance of an alternative oxidase increased by 49-fold, a superoxide dismutase by 15-fold and a major glutathione S-transferase by eight-fold. Expression remained elevated relative to Day 0 throughout 19 days of exposure to chilling, then declined substantially after 7 days of rewarming at 20°C. Interestingly, a catalase did not show this trend and was down-regulated during cold storage. Genes encoding a large number of heat shock, DNAJ and molecular chaperone proteins were also up-regulated (Figure 5B). Some of these increased to a maximum mRNA abundance after 1 day, others peaking at later times, but most remained at elevated abundance throughout the 19 days of chilling. A smaller number of similar proteins were down-regulated during exposure to chilling, suggesting that these were not directly involved in abiotic stress response. Upon transfer of the fruit to 20°C for 7 days, almost all the genes reverted to a transcript abundance similar to that prior to chilling exposure.

### Transcripts Encoding Genes Related to Cell Wall Modification and Stress Amelioration Respond Differently to Mild and Severe Chilling

**FIGURE 6:**
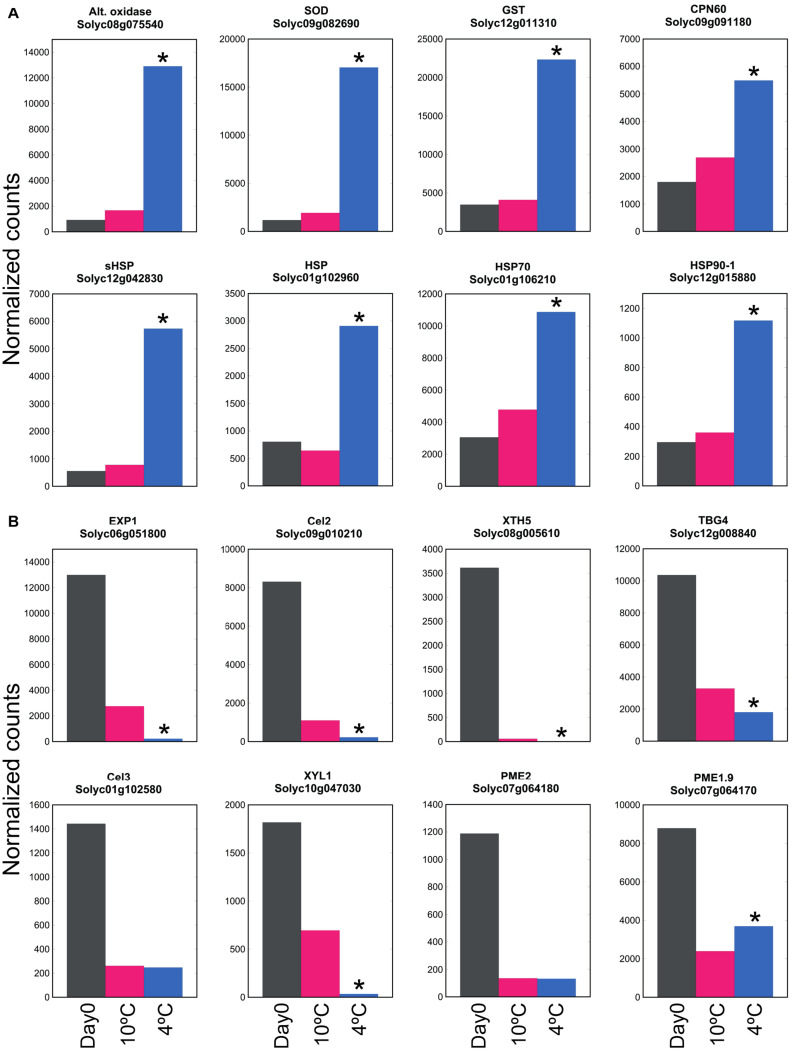
Effect of exposure to mild or severe chilling temperature on transcript abundance of genes related to stress and ripening. **(A)** Oxidative and temperature stress. Genes encoding antioxidant enzymes, chaperonins and HSPs. **(B)** Genes related to cell wall modification. Fruit were exposed to 10 or 4°C for 6 days, and transcript abundance determined using RNA-Seq. Asterisks denote that transcript abundance at 4°C was significantly different to that at 10°C at *P* < 0.05.

By comparing transcript abundance with that at Day 0 (rather than comparing 20, 10, and 4°C together at 6 days as shown in Figure 2B), the large number of genes up- or down-regulated at 4°C could be contrasted with the smaller number up- or down-regulated at 10°C (Supplementary Table 4). This showed that the antioxidant-related, HSP and chaperonin genes that were up-regulated at 4°C were not substantially up-regulated at 10°C (Figure 6A). In contrast, this comparison also showed that the majority of cell wall-related genes that were down-regulated at 4°C were also strongly down-regulated at 10°C (Figure 6B). Thus, cell wall-related gene expression (part of ripening) was sensitive to mild chilling temperatures of 10°C, but stress responses were not activated until a lower temperature was reached, such as 4°C.

### Transcripts Showing Large Responses to Chilling Stress

**FIGURE 7:**
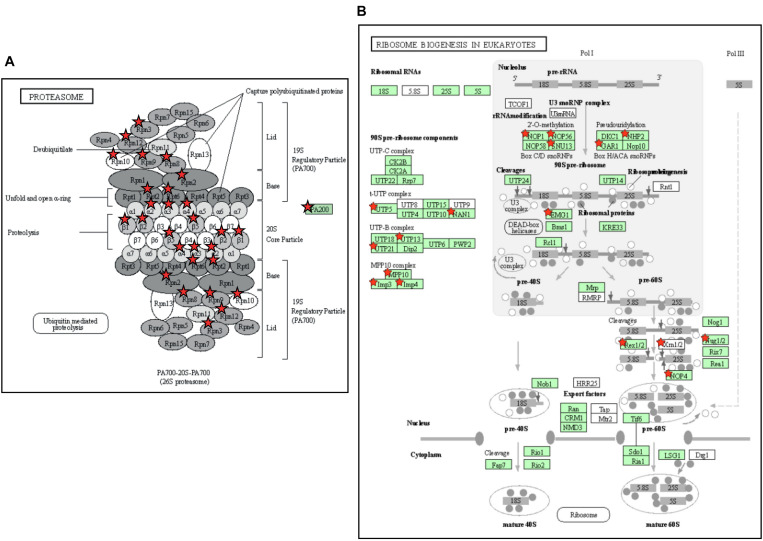
Enrichment of pathways related to protein turnover in tomato fruit stored at 4°C. **(A)** The Proteasome pathway. The pathway was 10.3-fold enriched, with a *P*-value of 1.0e-11 and a False Discovery Rate of 7.4e-10. **(B)** The Ribosome Biogenesis in Eukaryotes pathway. The pathway was 6.2-fold enriched, with a *P*-value of 6.8e-9 and False Discovery Rate of 2.5e-7. Enriched KEGG pathways were identified using DAVID with 804 genes that exhibited a >2-fold increase in expression at both 4 and 12 days of storage relative to Day 0. Genes present in the gene set are highlighted with a red star. The gene set can be found in Supplementary Table 5. (To save space, panel A has been edited by moving the red stars from a separate table onto the proteasome cartoon. The original figure is shown in Supplementary Figure 5).

The above two experiments have used different comparisons to identify cold-responsive genes, comparing the transcriptome of chilled fruit with that of fruit held for an equivalent time at a shelf temperature of 20°C, or to the transcriptome at Day 0 prior to cold exposure. In both cases, the genes identified as differentially expressed in response to cold were largely similar. The DAVID Gene Functional Classification Tool (Huang et al., 2009) was used to identify metabolic pathways highly enriched by exposure to chilling. The two most highly enriched pathways were “Proteasome” and “Ribosome biogenesis in eukaryotes” (Figure 7 and Supplementary Table 5). The enrichment of pathways in protein degradation and biosynthesis suggests a strong metabolic re-alignment towards altering the protein composition of the cells in order to alleviate the abiotic stress.

**FIGURE 8:**
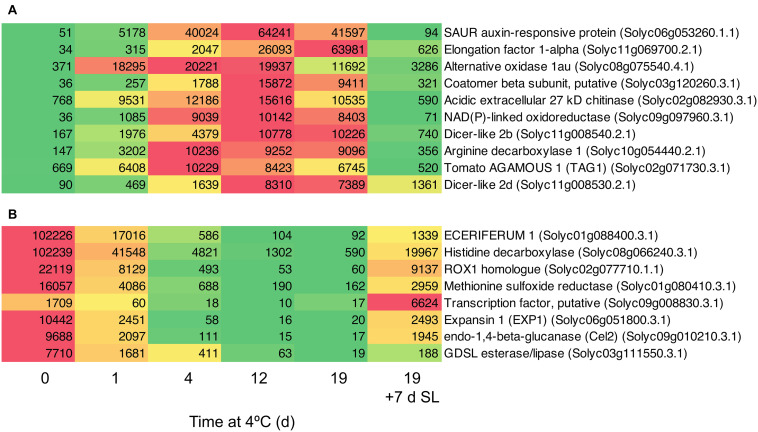
Heat maps showing genes strongly differentially expressed in response to storage at 4°C. An additional 7-day period at a shelf-life (SL) temperature of 20°C was also included after 19 days. **(A)** Transcripts showing very large fold changes up in response to temperature. **(B)** Transcripts showing very large fold changes down in response to temperature. Red and green colors represent high and low mRNA abundance, respectively, within each transcript. Numbers in the boxes are DESeq2 mean normalized counts using the median of ratios method.

Based on a comparison between Day 0 and 4 days of exposure to chilling, a number of genes showing the greatest fold increases (Figure 8A) or decreases (Figure 8B) were identified. Among the genes showing the greatest fold increases were a SAUR auxin-responsive family protein (increasing >1000-fold between 0 and 12 days), an alternative oxidase, two Dicer-like ribonucleases and the ripening-related transcription factor *TAG1*. The most down-regulated genes included *ECERIFERUM1*, a *ROX1* homolog (a transcription factor thought to act as a repressor of genes involved in hypoxia), and *EXP1* and *Cel2*. The data show that the expression of some genes is very highly responsive to chilling temperatures, and provide interesting avenues for further research.

### Validation of Transcript Abundance Determinations

Validation of transcript abundances was performed by repeating each RNA-Seq experiment on a different tomato cultivar. Transcript abundance data for eight target genes described in this study and four reference genes is presented in Supplementary Figure 6. These data show that trends and transcript abundances were repeatable in different RNA-Seq experiments, across two years, in three different cultivars, each with three biological replicates.

## Discussion

The manifestation of chilling injury symptoms varies depending on cultivar, developmental stage and the temperatures experienced by the fruit during chilled storage. Cherry tomatoes harvested at the breaker stage and stored at 4 or 5°C for 28 days displayed surface pitting and strongly impaired color development (and by inference ripening), which could not be reversed by an additional period of rewarming at 20°C (Albornoz et al., 2019; Zhang et al., 2019). In our experiments we subjected fruit which were already fully ripe to extended times at chilling temperature, with only slight effects on color and no observable surface pitting. However, textural failure is one of the symptoms of chilling injury in tomato fruit, and can include reduced softening, accelerated softening and the development of mealiness (Jackman et al., 1992; Marangoni et al., 1995; Devaux et al., 2005). We found that ripe ‘Angelle’ fruit stored at 4°C softened more than fruit stored at 10°C (Figure 1B) and developed a degree of mealiness after transfer to 20°C (Figure 1C).

In contrast, mature green ‘Caruso’ fruit (a large loose cultivar) stored at 5°C for 28 days were firmer than non-chilled fruit (Jackman et al., 1992), although Marangoni et al. (1995) found that they became softer than non-chilled controls if at 15 days the cold-stored fruit were transferred to 22°C for 10 days. This group also found that the middle lamella of chilled fruit was swollen and less defined, and it was suggested that water moving from the symplast to the apoplast was absorbed by the swollen middle lamella, reducing cell turgor and contributing to chilling-induced softening (Marangoni et al., 1995). From a study comparing storage at two chilling temperatures, Biswas et al. (2014) concluded that storage at 6°C mainly induced loss of turgor, whereas storage at 2.5°C caused loss of both turgor and tissue integrity. An interesting observation was that fruit stored at 10°C became slightly firmer during storage (Figure 1B), although the fruit subsequently softened when transferred to 20°C. In cranberries stored under controlled atmosphere and in blueberries, firmness was also reported to increase during storage in still air (Gunes et al., 2002; Paniagua et al., 2013), possibly related to firming of the outer cell layers due to localized dehydration during low water loss, while high water loss was associated with softening (Paniagua et al., 2013).

Mealiness is associated with a lack of juiciness, and a soft, grainy, dry unpleasant taste that is lacking in aroma. In stonefruit, it cannot be detected from outside the fruit by measuring firmness (Lurie and Crisosto, 2005). In peach and apple, mealiness was correlated with reduced intercellular adhesion and water-soluble pectin, and with reduced activity or expression of many enzymes including PG and PME (Brummell et al., 2004; Billy et al., 2008; Segonne et al., 2014). Expression of genes encoding both of these enzymes was reduced by chilling in tomato (Figure 4). Since we found chilling of the fruit was accompanied by greater softening, it seemed surprising that within 4 days many of the genes associated with softening were down-regulated by chilling stress. However, since storage at 10 or 4°C caused a similar down-regulation of expression of genes involved in cell wall modification (Figure 6B) but had different effects on fruit softening (Figure 1B), this is consistent with severe chilling conditions also affecting factors other than cell wall modification, such as turgor.

Of 11 cell wall-related genes showing altered expression during chilling, 10 were down-regulated and exhibited some degree of up-regulation when the fruit were transferred to shelf temperature (Figure 4). For the majority of these genes the effects of chilling were only partially reversible, and transcript abundances after rewarming of the fruit were only a fraction of the Day 0 value. This suggests that any softening occurring during rewarming is due to a very limited resumption of cell wall disassembly caused by increased cell wall-related gene expression. In the cases of *PG2a, PL, EXP1, TBG3*, and *Cel2*, this was to less than 25% of the Day 0 value. Comparing mRNA abundance after an additional 7 days at 20°C with that at 19 days under chilling temperature conditions, the largest increases in fold terms were in *XTH5* (764-fold), *EXP1* (125-fold) and *Cel2* (114-fold). However, these increases were from a very low base and mRNA abundances of *EXP1* and *Cel2* were still only 25% that of the Day 0 value. Of these three genes, so far only *EXP1* has been shown to have a measurable effect on fruit softening (Brummell et al., 1999a, b; Saladié et al., 2006). More recent work has shown that the *PL* gene product is important in tomato fruit softening (Uluisik et al., 2016; Yang et al., 2017). *PL* mRNA abundance was high in ripe fruit at Day 0, and declined by ∼7-fold to a relatively constant abundance during 4–19 days of storage at 4°C (Figure 4). Upon transfer of the fruit to 20°C, *PL* mRNA abundance increased but by less than two-fold and also to only one-quarter of the Day 0 value.

Comparisons between studies using different cultivars, developmental stages and temperature regimes can be problematic, but using an *S. pennellii* – *S. lycopersicum* introgression line harvested at breaker and stored at 3°C for 14 days, Rugkong et al. (2011) observed limited recovery of mRNA abundance during subsequent rewarming for *PG2a*, *TBG4*, and *EXP1*, but not for *XTH5* or *PE1*. In mature green ‘Micro-Tom’ fruit, storage at 4°C for 7 days reduced mRNA abundances of *PME1, Cel1, EXP1*, *TBG4*, and *XTH5*, and expression of all of these recovered to some extent after transfer to 20°C for 2 days (Müller et al., 2013). However, 7 days at 4°C may not be sufficient to induce permanent chilling injury.

One of the earliest responses to chilling was a large increase in the transcript abundance of enzymes involved in combating oxidative stress. Within 1 day of exposure to 4°C, substantial increases in the transcript abundance of an alternative oxidase, superoxide dismutase, and several glutathione S-transferases were observed (Figure 5A). The induction of a robust antioxidant system (Malacrida et al., 2006; Gómez et al., 2009; Vega-Garcia et al., 2010) and particularly alternative oxidase have been correlated with resistance to chilling injury in tomato as well as in pepper and banana (Fung et al., 2004, 2006; Albornoz et al., 2019; Hao et al., 2019). Upon rewarming of the fruit after severe chilling, transcript abundance changes in oxidative stress genes were almost entirely reversible (Figure 5A).

The small HSP Solyc12g042830.2.1 and the DNAJ domain-containing protein Solyc02g014860.3.1 were also up-regulated >10-fold within 1 day (Figure 5B). This was followed by slower increases in transcripts encoding a number of other HSPs and molecular chaperones, which remained elevated throughout chilling exposure. Upon rewarming of the fruit after severe chilling, transcript abundance changes in chaperonin-type genes were almost entirely reversible (Figure 5B). Strong cold-induced up-regulation of genes encoding numerous glutathione S-transferases and HSPs was also observed in ‘Micro-Tom’ (Cruz-Mendívil et al., 2015), a relatively chilling-tolerant cherry tomato line used for experimental studies (Luengwilai et al., 2012). A relationship between accumulation of small HSPs and resistance to chilling injury has been established in tomato (Sabehat et al., 1996, 1998), and appears to be common to many other species (Aghdam et al., 2013; Kashash et al., 2019). Small HSPs are believed to have a molecular chaperone function, stabilizing membranes and preventing proteins from mis-folding during heat, cold or oxidative stress (Sun et al., 2002). Comparisons of lines differing in chilling tolerance found that up-regulation of specific protein isoforms or transcripts occurred in chilling-tolerant cultivars, showing that only a few small HSPs contribute to better chilling resistance (Page et al., 2010; Ré et al., 2017). Similarly, in cold-stressed ripe tomato fruit that were analyzed prior to the symptoms of chilling injury becoming apparent, only certain HSPs and a single glutathione S-transferase showed increased protein abundance (Sanchez-Bel et al., 2012).

Cold storage of green or breaker tomato fruit down-regulates many pathways including those related to the ripening process (Rugkong et al., 2011; Zhang et al., 2019; Tang et al., 2020), but in the ripe fruit cold-affected transcriptome we detected two pathways that were strongly up-regulated. These were both related to protein metabolism: a proteasome pathway involved in protein degradation and a ribosome biogenesis pathway (Figure 7). In chilling-sensitive ‘Heinz-722’ mature-green fruit stored at 5°C, observations by electron microscopy showed that after 7 days the endoplasmic reticulum became dilated and ribosomes were lost, while membranes appeared degraded (Marangoni et al., 1989). In ripe ‘p73’, an early effect of cold stress (prior to symptoms becoming evident) was a down-regulation of the protein degradation machinery (Sanchez-Bel et al., 2012). These observations suggest that protein metabolism is an early casualty of cold stress. The subsequent up-regulation of pathways involved in ribosome biogenesis and protein degradation could be responses by the cell to counteract these chilling-induced degenerative processes.

Genes in Clusters 1 and 5 (Figures 3C,D) were highly responsive to temperature. In ‘Micro-Tom’, the rapid up-regulation of a dehydrin transcript has been proposed as a molecular marker of cold stress (Weiss and Egea-Cortines, 2009). The accumulation of HSPs has been proposed as a biochemical marker of chilling stress (Aghdam et al., 2013), and increased abundance of the corresponding *HSP* transcripts could also be used as molecular markers. However, we have identified genes with much greater increases in response to chilling stress than HSPs, particularly genes encoding a SAUR auxin-responsive protein, elongation factor 1-alpha, alternative oxidase, and a putative coatamer beta-subunit (Figure 8A). These showed massive increases in transcript abundance relative to Day 0 (the first two >1000-fold), with maxima at different times of chilling exposure. It may be possible to use these highly temperature-responsive transcripts for screening tomato lines for the timing and extent of responses to chilling stress. There may also be biochemical insights to be gained from the strong up-regulation of the *SAUR* gene in response to cold. The functions of SAUR proteins remain mainly elusive, but *SAUR* genes are responsive to abiotic stress and the proteins can interact with other hormonal pathways (Ren and Gray, 2015). In Arabidopsis, up-regulation of *AtSAUR32* regulates ABA-mediated responses to drought stress (He et al., 2021), and in chilled tomato fruit up-regulation of a *SAUR* may indicate the involvement of auxin pathways in cold response or chilling injury onset, which have previously been associated mainly with ethylene.

In conclusion, chilling injury in ripe ‘Angelle’ fruit was relatively modest even after extended storage at 4°C, consisting of slight color loss, accelerated softening and the development of mealiness. Decreases in the transcript abundance of many genes involved in cell wall disassembly, and the expected low activities of enzymes operating at 4°C, suggest that accelerated softening at such chilling temperatures was due to factors other than cell wall metabolism, such as reductions in turgor. Chilling-induced decreases in the expression of cell wall disassembly-related genes were only partially reversible upon rewarming of the fruit. Responses to cold stress included a rapid increase in the transcript abundance of several antioxidant-related genes involved in protection of the cell from oxidative damage, followed by large increases in HSPs and molecular chaperones. These increases were specific to 4°C, not being observed at 10°C, and were reversible when the fruit were rewarmed to a 20°C shelf temperature. Expression of pathways of genes involved in protein synthesis and degradation were also increased at 4°C, probably as the fruit responded to chilling-induced damage to the proteome.

## Data Availability Statement

The datasets presented in this study can be found in online repositories. The names of the repository/repositories and accession number(s) are: SRA NCBI, accession: PRJNA736844.

## Author Contributions

DH, RL, and DB conceived and planned the study. DH and NN carried out RNA-Seq data analysis and prepared the figures. ZE and NN isolated RNA, AS created RNA-Seq analysis software. DH, NN, ZE, RC, MM, EO’D, MH, RL, and DB carried out fruit physiology measurements. LF carried out RNA-Seq data analysis. DB wrote the manuscript. All authors contributed to the article and approved the submitted version.

## Conflict of Interest

The authors declare that the research was conducted in the absence of any commercial or financial relationships that could be construed as a potential conflict of interest.

## Footnotes

1 http://www.bioinformatics.babraham.ac.uk/projects/fastqc/

2 http://factominer.free.fr/citation.html

3 https://david.ncifcrf.gov/tools.jsp

4 https://plants.ensembl.org/biomart/martview/

5 https://www.genome.jp/kegg/pathway.html
