# Supplementary material for: Transcriptome Responses of Ripe Cherry Tomato Fruit Exposed to Chilling and Rewarming Identify Reversible and Irreversible Gene Expression Changes
Source: Front Plant Sci. 2021 Jul 16;12:685416. doi: 10.3389/fpls.2021.685416 (PMC8322768; doi:10.3389/fpls.2021.685416)
Supplement: Supplementary file 1 [file Data_Sheet_1.PDF]

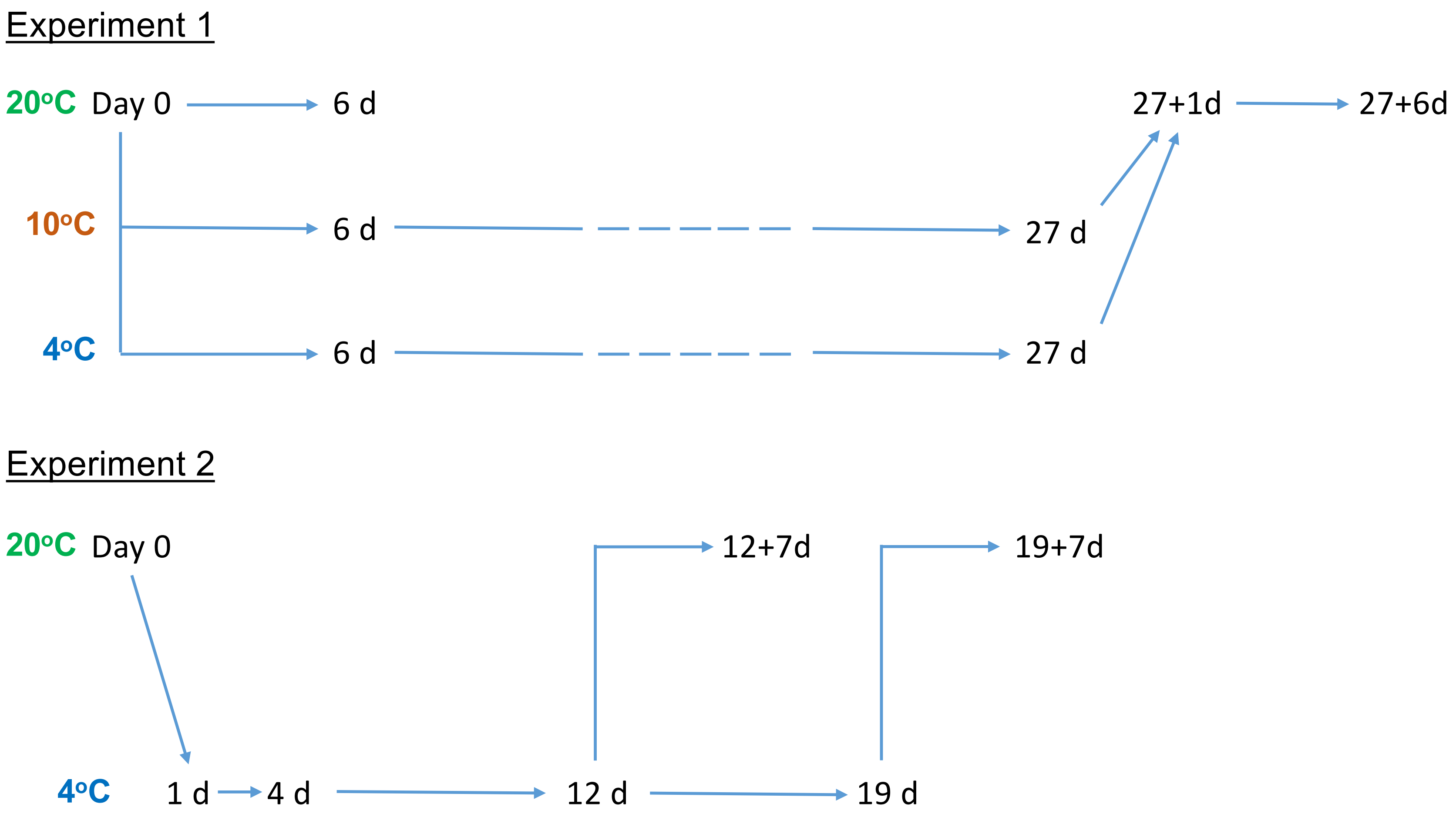

Supplementary Figure S1. Schematic representation of the two experiments. Experiment 1 was a comparison of storage at mild (10°C) and severe (4°C) chilling, whereas Experiment 2 focused on rapid transcriptome responses to severe chilling. After certain times at chilling temperatures, fruit were transferred to a shelf temperature of 20°C for 1, 6 or 7 d as indicated. Triplicated samples for RNA-Seq were taken at each time/temperature point (except Experiment 1, 27+6d, which was used just for physiological measurements).

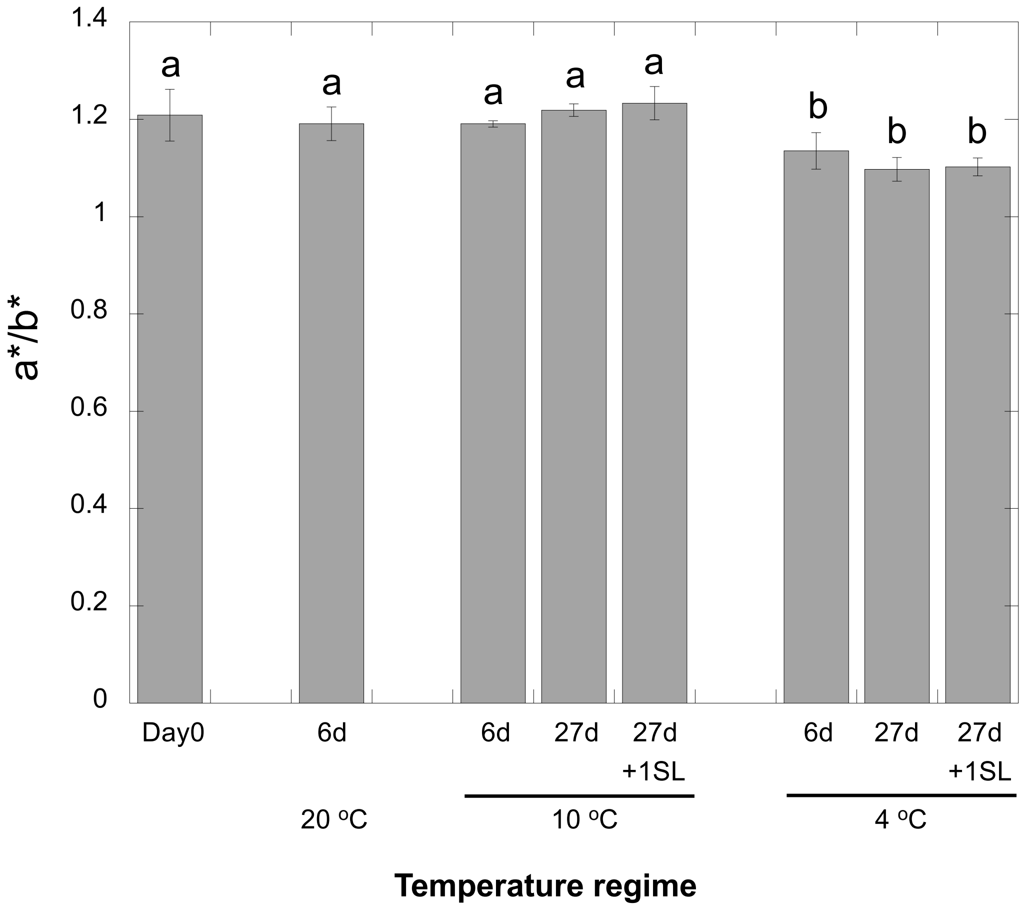

Supplementary Figure S2. Fruit color after storage at 20, 10 or 4°C for up to 27 d, followed in some cases by re-warming to shelf-life (SL) conditions of 20°C for 1 d. Fruit color was measured using the CIELAB color space L*a*b* system and expressed as a*/b* ratio. An increase in the ratio of a* (red-green) to b* (blue-yellow) indicates an increase in redness, assumed to be an indicator of ripening. The decreased redness of fruit stored at 4°C is a chilling injury possibly related to membrane damage in the chromoplasts. Data are means of three replicate punnets per time point ±SD. For each punnet, two readings were taken from each of eight different fruit (n = 48). Means not sharing a common letter are significantly different between groups at P = 0.05 as determined by LSD after a one-way ANOVA.

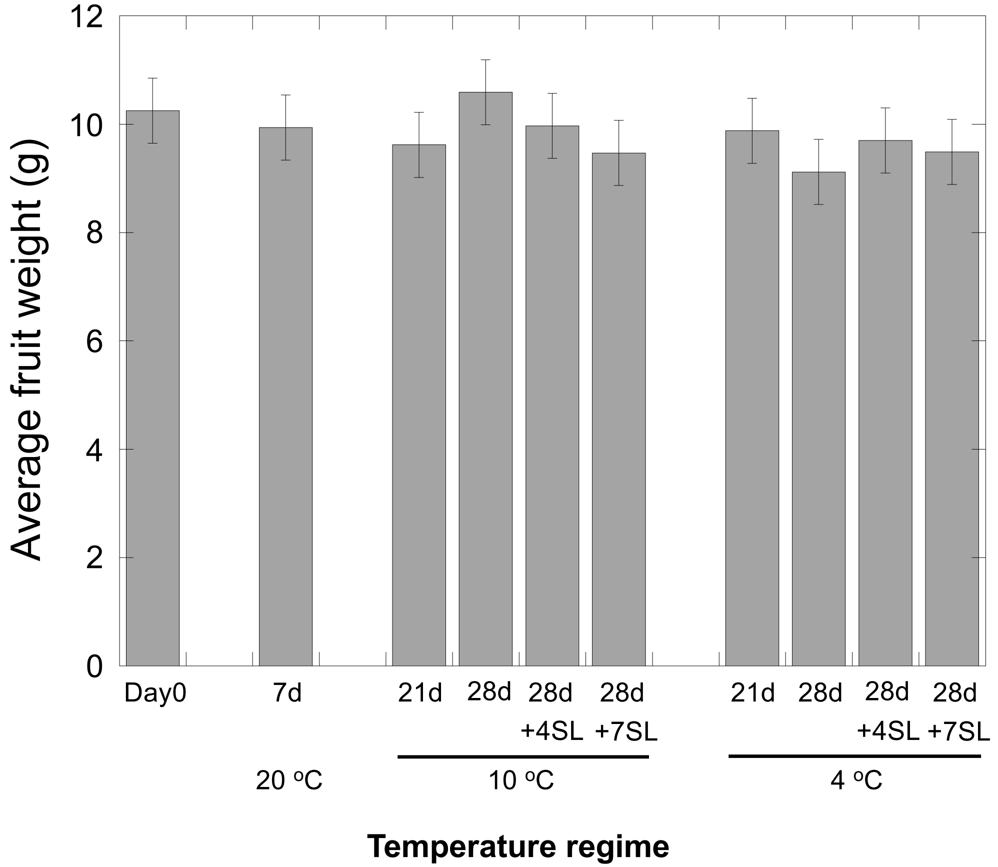

Supplementary Figure S3. Fruit weight after storage at 20, 10 or 4°C for up to 28 d, followed in some cases by re-warming to shelf-life (SL) conditions of 20°C for 4 or 7 d. Weight loss during cold storage is probably due mainly to moisture loss, whereas during shelf conditions some loss of dry matter also occurs. Data are means of three replicate punnets per time point (each punnet containing 20-23 fruit). Data were analysed by ANOVA and a least significant difference (P = 0.05) of 0.6 (indicated by the bars above each column) applies to all comparisons.

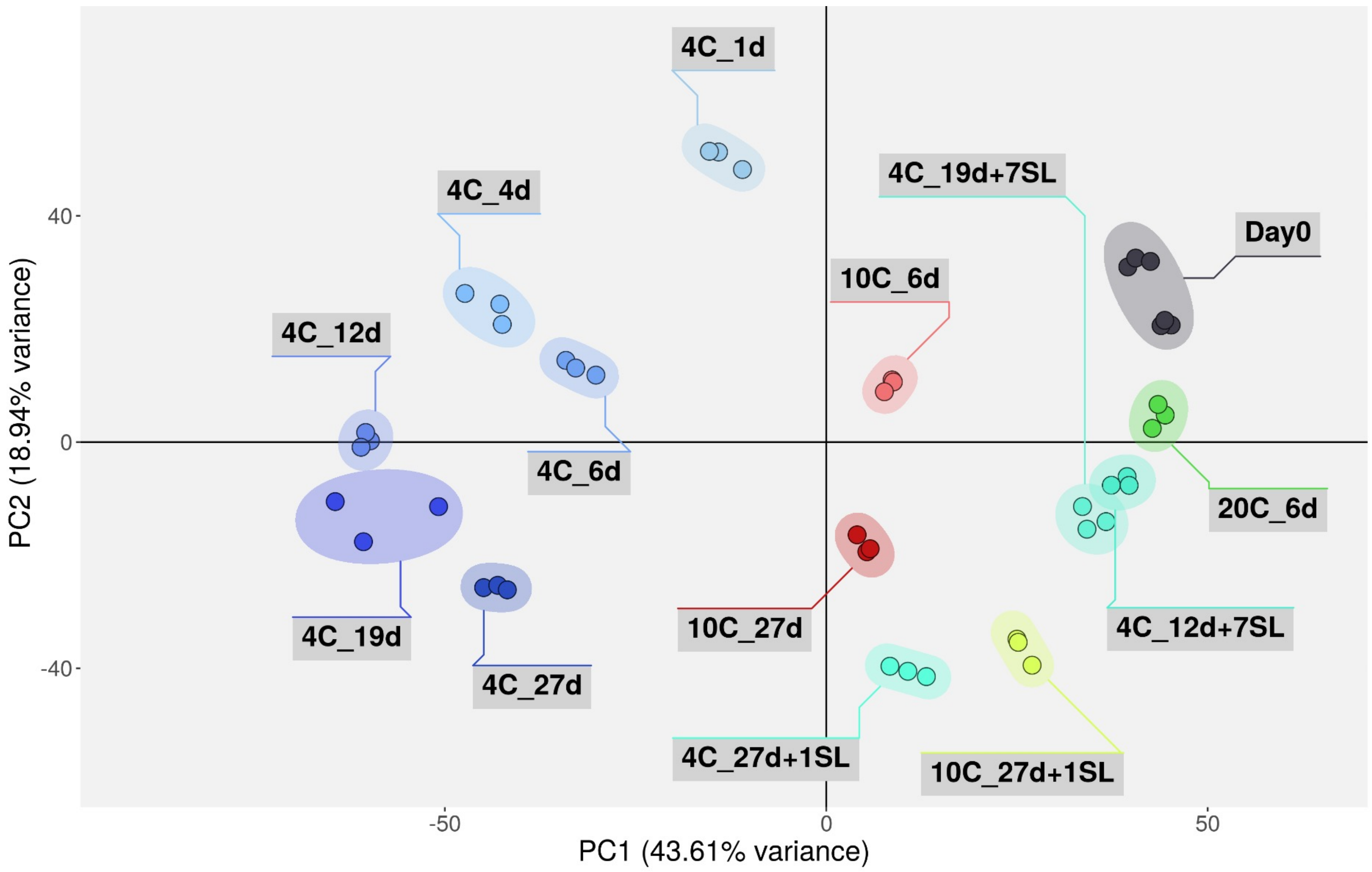

Supplementary Figure S4. Principal component analysis of transcriptome profiles showing combined data from the experiments of Figs. 2A and 3A. Comparisons are based on the 50 most variable genes in each pairwise comparison (total of 1133 most variable genes), showing changes during storage for various times at 20, 10 or 4°C, followed in some cases by a period of 1 or 7 d at shelf-life (SL) temperature of 20°C.

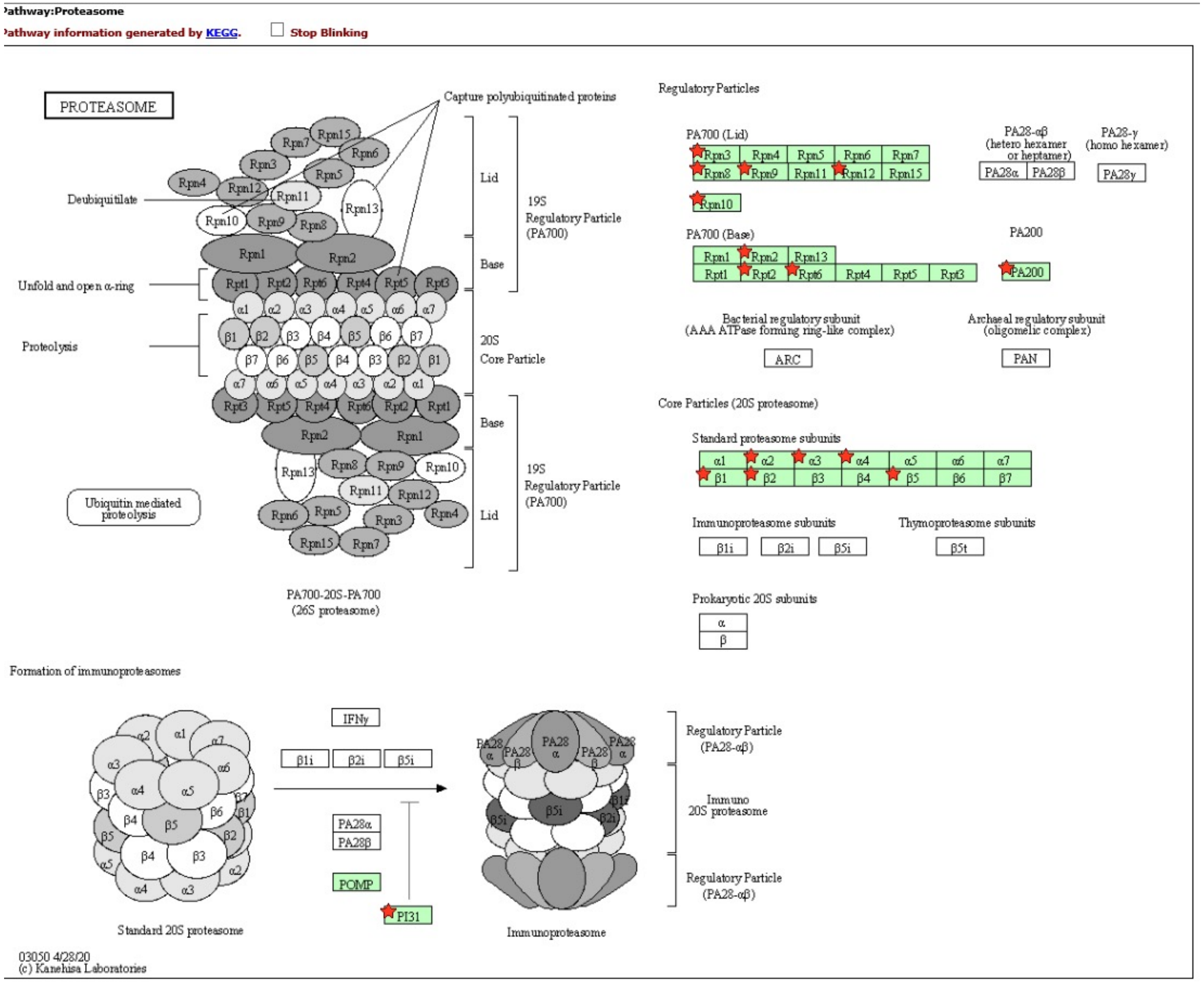

Supplementary Figure S5. Enrichment of the Proteasome pathway in tomato fruit stored at 4ºC. Enriched KEGG pathways were identified using DAVID with 804 genes that exhibited a >2-fold increase in fruit at both 4 and 12 d of storage relative to Day 0. The pathway was 10.3-fold enriched, with a False Discovery Rate of 7.4E-10. Genes present in the gene set are highlighted with a red star. The gene set can be found in **Supplementary Table S4**.

Experiment 1, 2018		cultivar 'Angelle'						cultivar 'SugaBabe'					
		Time 0			4C 6days			Time 0			4C 6days		
Target genes		BioRep1	BioRep2	BioRep3	BioRep1	BioRep2	BioRep3	BioRep1	BioRep2	BioRep3	BioRep1	BioRep2	BioRep3
Solyc06g053260.1.1	SAUR-like auxin-responsive family protein	23.7	95.7	19.6	32060.1	38023.3	39315.5	302.1	163.7	136.4	51884.5	37562.3	41142.2
Solyc06g051800.3.1	expansin 1 (EXP1)	13969.5	15297	15956.3	231.3	306.4	280.6	10374.4	9520.9	10672.2	281.4	260.5	192
Solyc09g010210.3.1	endo-1,4-beta-glucanase precursor (Cel2)	8866.6	11337.4	8694	218.6	324.7	287.7	9699.4	7660.3	8068.9	165.5	136.7	188.9
Solyc07g064180.3.1	pectin methylesterase 2	1345.2	1637.3	1147.4	208.3	152.2	140.9	610	529.8	725.8	61	55	87.3
Solyc08g075540.4.1	alternative oxidase 1au	1212.7	1084.4	888.8	15266.5	16755.4	17104.7	4546.1	3871.3	3505.4	27664.5	21983.5	22550.6
Solyc12g011310.2.1	Glutathione S-transferase	4171.8	3541.5	4350.7	26558.5	30957.8	27369.6	20193.2	13801	22808.3	41246.5	38609	47120.7
Solyc01g106210.3.1	Heat shock protein 70	3517.5	3512.5	3591.9	12989.3	14620.1	13741.5	6124.6	5678.9	6166.7	13005.8	14905.5	13392.4
Solyc07g047790.3.1	Heat shock protein, putative	2477.5	2502.5	2467.7	9412.8	9602.1	9349.2	4450.5	4347.4	4250.7	10299.7	10122.6	11191.1
Reference genes													
Solyc01g104775.1.1	Actin	2350.9	2353.9	2470.2	2123.1	2262.9	2269.6	2163.6	2147.7	2036.5	3124	3180.3	3012.4
Solyc11g005330.2.1	Actin2	12482.3	12081.7	13776.6	8918	9149.7	10189.7	10457.5	10426.8	10297.7	9004.6	8719.1	7735.2
Solyc03g115810.3.1	SAND family	493.4	527.7	485.5	303.8	213.4	256.7	462.7	544	529.1	279.3	304.5	331.6
Solyc08g006960.3.1	Clathrin adaptor complexes medium subunit	667.3	717.9	706.1	589.2	510.6	489.5	696	695.2	672.8	409.6	413.7	417.9

Experiment 2, 2019		cultivar 'Angelle'						cultivar 'Bambello'					
		Time 0			4C 12days			Time 0			4C 12days		
Target genes		BioRep1	BioRep2	BioRep3	BioRep1	BioRep2	BioRep3	BioRep1	BioRep2	BioRep3	BioRep1	BioRep2	BioRep3
Solyc06g053260.1.1	SAUR-like auxin-responsive family protein	61.2	43.5	49.4	68827.1	64493.3	63006.2	68.9	96.4	69.6	72308.2	66814.2	82825.4
Solyc06g051800.3.1	expansin 1 (EXP1)	8934	10149.2	11767.6	18.4	14	17.4	17700.6	16612.5	15619.1	25	34.9	20
Solyc09g010210.3.1	endo-1,4-beta-glucanase precursor (Cel2)	8457.5	10048.8	10137.6	15.9	21.8	8.7	11277.3	10965.8	12517.7	36.1	31.2	42.6
Solyc07g064180.3.1	pectin methylesterase 2	1525.5	1578.1	1544.4	55.3	46.3	27.1	1626.5	1563.5	1543.5	57.4	70.7	40
Solyc08g075540.4.1	alternative oxidase 1au	441	354.2	307.2	20081.4	22241.2	18623.9	1600.9	2343.4	1446.3	16194.1	14345.8	14662.5
Solyc12g011310.2.1	Glutathione S-transferase	3603.7	3660.6	3101.9	11116.4	15019.1	11803	2309.1	2526.5	1902	8738.7	7820.3	14348.3
Solyc01g106210.3.1	Heat shock protein 70	2299.9	2134.7	2240.3	10296.2	9886.6	10288.4	2334.6	2032	2549.4	9529.8	9972.8	8576.6
Solyc07g047790.3.1	Heat shock protein, putative	1841.6	1548.8	1798.9	6658.6	6806.3	6948.6	2042.7	1814.8	1984.9	6900.5	6997.5	7064.1
Reference genes													
Solyc01g104775.1.1	Actin	1394.8	1304.5	1338.4	1771	1790.9	2161	1493.7	1335.9	1470.4	2218.7	2915.6	2646.5
Solyc11g005330.2.1	Actin2	7398.5	7036.5	6208.7	5959.9	5922.2	6082.5	7563.9	9152.5	8158.2	7841.7	8351.1	7855.1
Solyc03g115810.3.1	SAND family	421.1	478.3	501.6	219.5	201.8	121.9	509.8	324.7	564.4	171.5	202.9	163.6
Solyc08g006960.3.1	Clathrin adaptor complexes medium subunit	447.6	510	490.9	251.3	307.5	251.6	450.2	499.7	419.1	307.6	256.2	307.2

Supplementary Figure S6. Validation of transcript abundances for selected genes used in this study. RNA-Seq determinations of transcript abundance were made using normalized counts from DESeq2, shown here repeated in two different years using three different cultivars. The RNA-Seq experiments were independent, using different sequencing providers, and had three biological replicates per time point. The expression of four reference genes is also shown. Reference genes were chosen to encompass low, moderate and more highly expressed genes and the latter three were taken from Gonzalez-Aguilera et al. (2016), who showed by RT-qPCR that these genes had stable expression across tomato fruit development. To help visualize differences between treatments, each row of the target genes was conditionally formatted for colour in Excel using a red/yellow/green colour scale and default parameters. For the reference genes, the four rows were conditionally formatted as one block in the red/yellow/green scale to highlight stability of their expression across the treatments.

Gonzalez-Aguilera, K.L., Saad, C.F., Chavez Montes, R.A., Alves-Ferreira, M., de Folter, S. 2016. Selection of reference genes for quantitative real-time RT-PCR studies in tomato fruit of the genotype MT-*Rg1*. Frontiers Plant Sci. 7, 1386.
